# Noble metal-based electrocatalysts for selective alcohol oxidation to high-value chemicals: from C–C bond retention mechanism to catalyst design

**DOI:** 10.1039/d6sc03223k

**Published:** 2026-05-27

**Authors:** Wenjing Zhang, Bingrong Wang, Jing Li, Zidong Wei

**Affiliations:** a College of Materials and Chemistry & Chemical Engineering, Chengdu University of Technology Chengdu 610059 P. R. China; b School of Chemistry and Chemical Engineering, Chongqing University Daxuecheng South Road 55 Chongqing 401331 China lijing@cqu.edu.cn zdwei@cqu.edu.cn

## Abstract

The electrocatalytic oxidation of biomass-derived alcohols has emerged as a sustainable route for green hydrogen production, offering a compelling alternative to conventional water electrolysis by replacing the energy-intensive oxygen evolution reaction with thermodynamically more favorable alcohol oxidation reactions. However, the practical application of this technology faces a critical scientific challenge arising from the competition between C–C bond retention and cleavage pathways. The cleavage pathway generates low-value products (*e.g.*, formic acid, CO_2_) and causes severe catalyst poisoning due to carbon-containing intermediate adsorption, whereas the retention pathway yields high-value aldehydes, ketones, or carboxylic acids. Achieving highly selective C–C bond retention is therefore essential for realizing the synergistic benefits of energy savings and product valorization. This review systematically explores recent research advances in selective alcohol oxidation to high-value chemicals, with an emphasis on the mechanism of C–C bond cleavage or retention pathways, as well as catalyst design based on noble metal materials. These design strategies collectively provide a solid foundation for achieving highly selective C–C bond retention in alcohol electrooxidation. With further advancements in this field, alcohol oxidation-assisted hydrogen production technology is expected to play an increasingly important role in future green hydrogen systems and biomass refining, contributing to the transition toward a sustainable and low-carbon chemical industry.

## Abstract 2

The electrocatalytic oxidation of biomass-derived alcohols replacing the oxygen evolution reaction has emerged as a sustainable route for green hydrogen production, which also conducive to the high-value products through C–C bond retention pathway.

## Graphical abstract

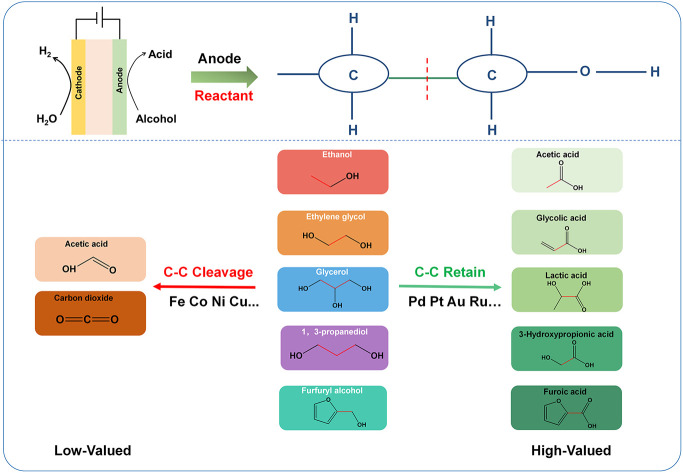

## 1 Introduction

Hydrogen energy, with its high energy density and zero-carbon emission advantages, is regarded as a key carrier for building a future clean and low-carbon energy system.^1–3^ However, the current mainstream hydrogen production processes are still heavily reliant on fossil fuel reforming, which not only consumes enormous energy but also emits large amounts of carbon dioxide throughout the entire supply chain, contradicting the principles of green and low-carbon development.^4^ In contrast, water electrolysis technology can efficiently produce high-purity hydrogen under ambient conditions, achieving zero carbon emissions and representing a core pathway for green hydrogen production. From an economic perspective, electricity costs account for 60–80% of the total cost of water electrolysis, making it a key factor determining economic viability.^5–7^ Although the declining cost of renewable energy has gradually brought water electrolysis closer to conventional hydrogen production costs, current technologies still face severe bottlenecks, where the high overpotential of the anodic oxygen evolution reaction (OER) leads to high electricity consumption. Meanwhile, the low-valued oxygen anodic product makes it difficult to offset costs, covering only 5–10% of the total expenditure.^8^ More importantly, the difficulty in completely isolating oxygen from hydrogen poses explosion likelihood, further increasing system safety costs.^9,10^ These characteristics of high energy consumption and low return accompanied by safety concerns significantly constrain the large-scale industrial application of water electrolysis. Therefore, developing new electrocatalytic systems that balance efficiency, safety, and economy has become a central challenge in the field of green hydrogen research.

To address the energy consumption and safety bottlenecks of water electrolysis, researchers have proposed replacing the anodic OER with the thermodynamically more favorable oxidation of biomass-derived alcohols.^11–13^ These substrates are widely available and inexpensive, with oxidation potentials significantly lower than that of OER, enabling efficient hydrogen production at lower cell voltages and thus reducing system electricity consumption.^14–16^ Moreover, alcohol oxidation is often accompanied by the generation of high-value chemicals, which significantly improves overall process economics.^17^ In addition, alcohol oxidation inherently avoiding the safety risks associated with hydrogen–oxygen mixing. However, the practical application of alcohol oxidation still faces critical scientific challenges, such as the multi-electron transfer nature leads to sluggish reaction kinetics, the high overpotentials that partially offset the advantage of low thermodynamic potentials.^18^ More importantly, the complexity of multi-electron transfer pathways results in a wide variety of reaction intermediates, with product spectra encompassing aldehydes, ketones, carboxylic acids, and even CO_2_, making precise control of product selectivity difficult.^19^ Taking glycerol as an example, the C–C bond cleavage pathway generates low-value formic acid, whereas the C–C bond retention pathway yields high-value dihydroxyacetone or glyceric acid. Low selectivity not only undermines the economic advantage of offsetting hydrogen production costs with high-value products but can also lead to poisoning of active sites by intermediates, affecting long-term operational stability.^20–22^ Therefore, how to effectively regulate the reaction pathway to achieve C–C bond retention while maintaining the energy-saving benefits of alcohol oxidation has become a scientific challenge in this field.

Rational catalyst design is considered an approach to overcoming the bottlenecks of biomass alcohol oxidation. An ideal catalyst should achieve synergy in three aspects. Firstly, it should efficiently activate C–H and O–H bonds in alcohol molecules by optimizing the electronic structure and coordination environment of active centers at the kinetic level, lowering the energy barriers of key elementary steps to drive multi-electron transfer processes efficiently at low overpotential.^23,24^ Secondly, it should precisely steer the reaction pathway toward target products at the selectivity control level, requiring well-defined active site configurations on the catalyst surface to stabilize specific reaction intermediates and suppress C–C bond cleavage or side reactions.^25,26^ Thirdly, it should possess good resistance to poisoning and structural stability, withstanding strong adsorption of reaction intermediates and oxidative corrosion under industrial high-current-density conditions at the stability level.^27,28^ Thus, an ideal catalyst should strike a balance among high activity, high selectivity, and high stability, achieving the synergy of these three highs. In recent years, researchers have conducted extensive explorations around these objectives, making significant progress in the structural optimization of noble metal-based catalysts, activity enhancement of non-noble metal catalysts, and interface engineering of carbon-based composites, laying a solid foundation for the development of efficient and stable biomass alcohol oxidation catalysts.

**Fig. 1:**
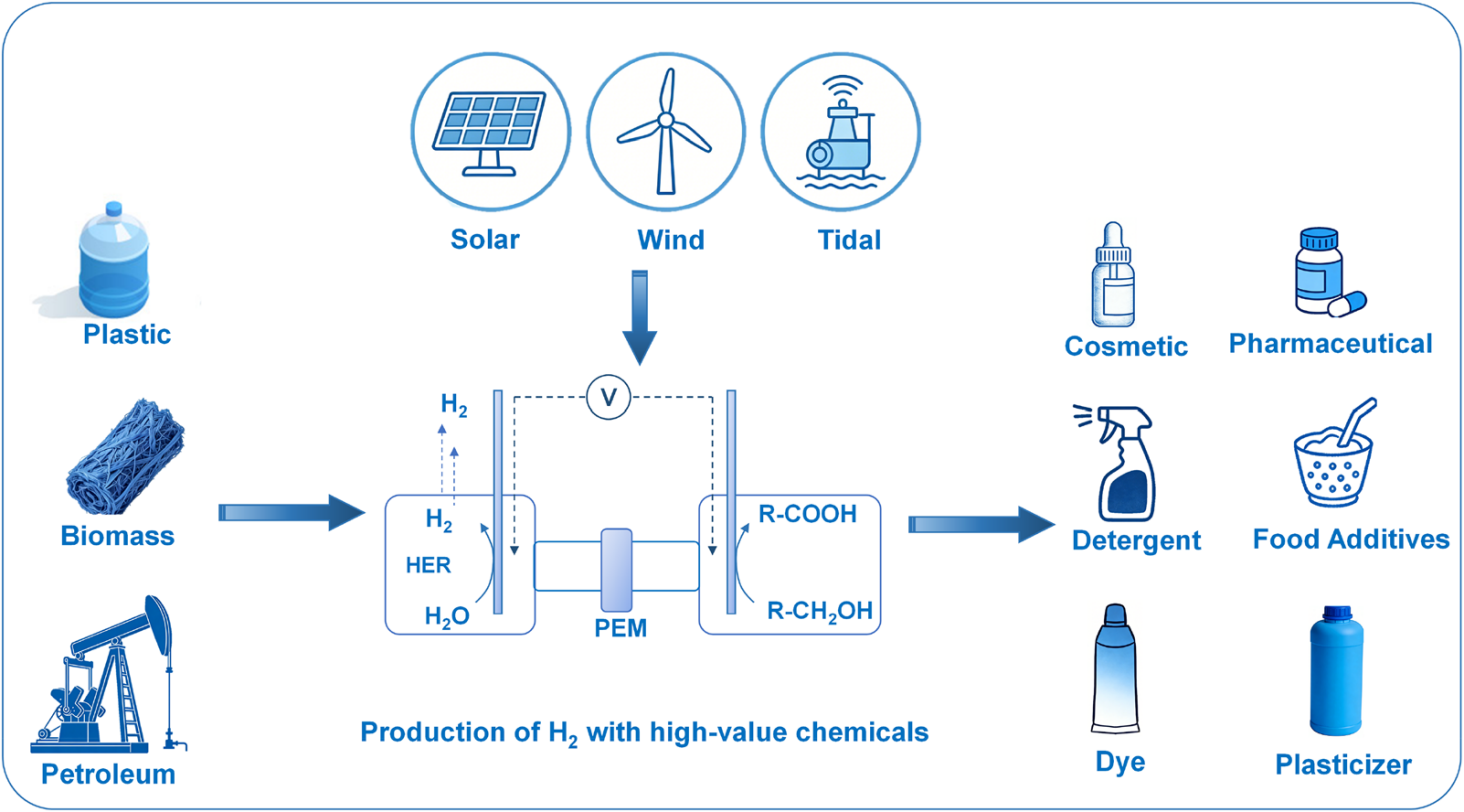
The illustration of electrocatalytic upgrading of carbon resources based on the classification to generate commodity chemicals.

Although several reviews have discussed catalyst design or reaction pathways for alcohol electrooxidation,^29–32^ few have systematically integrated the entire chain from catalytic mechanism to material design from the economic perspective of C–C bond retention for product valorization. In this review, we focus on the electrochemical oxidation of low-value alcohol molecules derived from biomass and plastics to obtain high-value additional products through alcohol oxidation reactions (Fig. 1). Based on the analysis of the competitive mechanisms between C–C bond cleavage and retention, we systematically summarize the design principles and representative examples of four advanced catalyst architectures-single atom catalysts, monometallic metal materials, alloys and composite materials for regulating C–C bond retention selectivity. Finally, we provide a brief summary and offer an outlook on the field of electrocatalytic production of high-value chemicals. Through this review, we aim to provide guidance on the design of functional electrocatalysts and the development of application-oriented engineering strategies, ultimately offering viable solutions for the sustainable electrocatalytic valorization of biomass and plastics.

## 2 Reaction mechanism: C–C bond cleavage and retention

The electrochemical oxidation of alcohols involves multi-step electron transfer and proton-coupled processes, with complex reaction networks and product distributions highly dependent on catalyst surface properties, applied potential, pH, and substrate structure.^33–36^ Understanding the competitive mechanism between C–C bond retention and cleavage is a theoretical prerequisite for moderate selectivity. This part will elaborate on the alcohol oxidation mechanisms, the molecular mechanisms of C–C bond cleavage pathways, and the molecular mechanisms of C–C bond retention pathways, laying the theoretical foundation for catalyst design.

### 2.1 Alcohol oxidation mechanisms

When noble metals serve as catalytic materials, their surface atoms chemically adsorb alcohol molecules through electronic interactions (*e.g.*, d-orbital overlap), serving as active centers for the oxidation reaction.^37^ The electrocatalytic oxidation of alcohols can be divided into the following steps. Firstly, alcohol molecules diffuse from the electrolyte to the electrode surface. Then, the alcohol molecules dehydrogenate and adsorb onto the electrode, with the adsorption configuration directly influencing subsequent reaction pathways, where the end-on adsorption *via* oxygen atoms facilitates subsequent dehydrogenation and side-on adsorption *via* the carbon chain may increase the likelihood of C–C bond cleavage. Subsequently, the adsorbed alcohol molecules undergo dehydrogenation on the catalyst surface, gradually losing protons and electrons, which is the rate-determining step of alcohol oxidation. Electron transfer occurs at the electrode surface, generating corresponding oxidation products, which finally desorb from the electrode surface and diffuse into the bulk solution.^38^

Different alcohols exhibit significant differences in oxidation pathways and C–C bond cleavage tendencies due to variations in carbon chain length, hydroxyl position, and the presence of other functional groups or substituents.^39^ For primary alcohols such as ethanol and *n*-propanol, oxidation of the terminal hydroxyl group generates an aldehyde intermediate, which is highly prone to decarbonylation on noble metal surfaces, leading to a high likelihood of C–C bond cleavage.^19,40^ In contrast, secondary alcohols such as isopropanol and 2-butanol oxidize directly to ketones, whose carbonyl groups are flanked by alkyl groups, making the C–C bond more stable and less prone to cleavage, thus exhibiting higher C–C bond retention selectivity.^41^ In polyol alcohols, vicinal diol structures such as glycerol and ethylene glycol (EG) are prone to C–C bond cleavage under alkaline conditions.^42^ In contrast, non-vicinal polyols such as 1,3-propanediol, with hydroxyl groups separated by a carbon atom, lack such synergistic effects and exhibit significantly lower cleavage tendencies.^43^ Aromatic alcohols such as benzyl alcohol and furfuryl alcohol, due to the conjugation of the resulting aromatic aldehydes with the benzene ring, have reduced carbonyl carbon electrophilicity and significantly increased decarbonylation activation energy, effectively suppressing C–C bond cleavage and serving as ideal model substrates for studying C–C bond retention mechanisms.^44,45^ Different metals exhibit distinct adsorption configurations and strengths during alcohol oxidation due to differences in their electronic structures, directly influencing catalytic performance and reaction pathways. This characteristic provides both abundant possibilities and challenges for the design of electrocatalytic alcohol oxidation processes, motivating researchers to continuously explore and optimize catalytic materials to achieve more efficient alcohol oxidation.

**Fig. 2:**
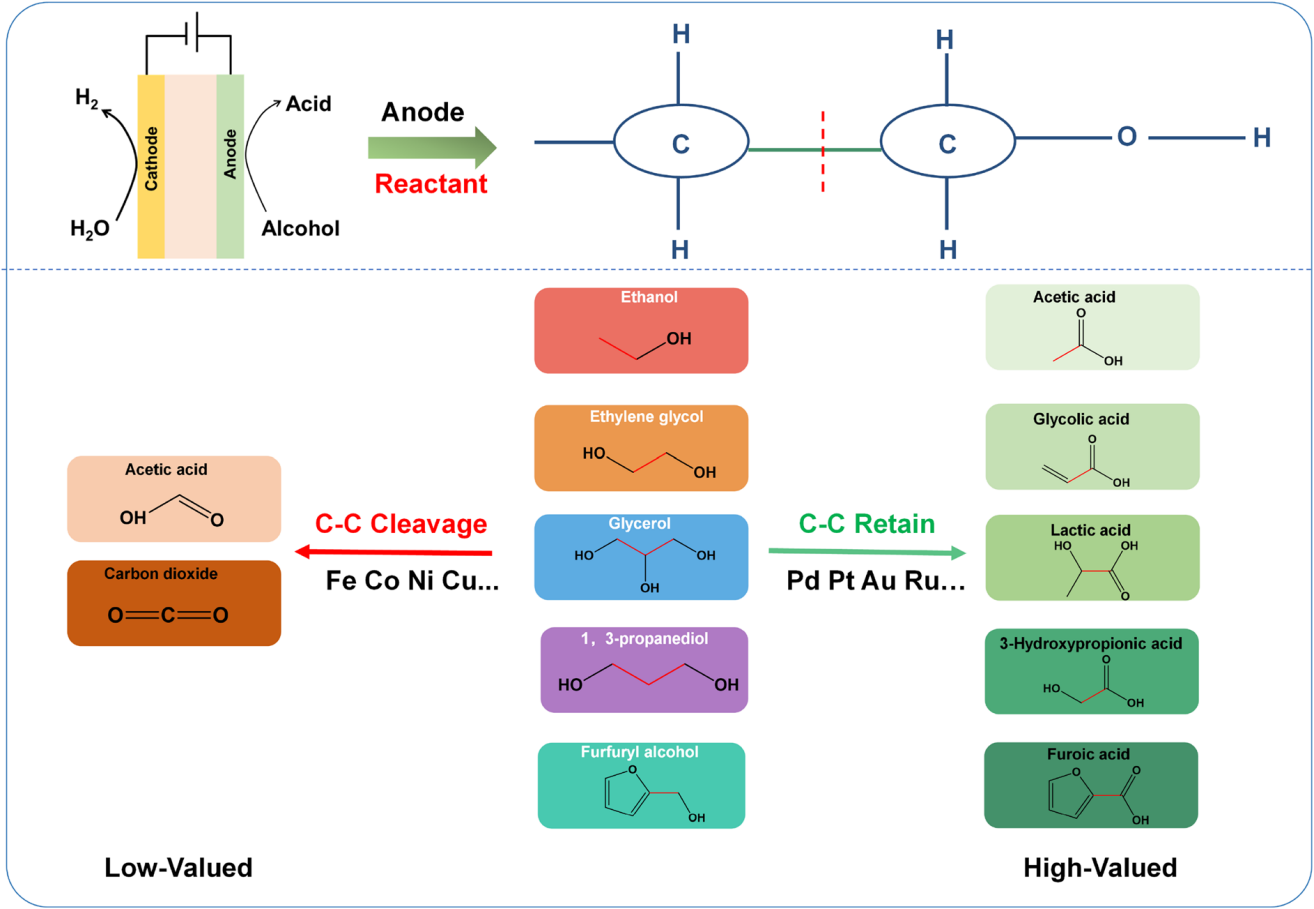
Electrocatalytic upgrading of alcohols: reactants and products in the C–C bond-retaining and bond-cleavage pathways.

From the above alcohol oxidation process, whether the C–C bond is retained serves as the branching point of the entire reaction pathway, and the direction of this branch directly determines the product value and the sustainability of the catalytic process. In the C–C bond retention pathway, the aldehyde intermediate desorbs from the surface into the solution or is attacked by nucleophiles to form carboxylic acids, maintaining an intact carbon chain and high product value. In the C–C bond cleavage pathway, the aldehyde intermediate undergoes decarbonylation on the catalyst surface, generating carbon monoxide and hydrocarbon fragments, which not only reduces product value but also causes catalyst deactivation due to carbon monoxide poisoning (Fig. 2). Therefore, understanding the molecular mechanisms of C–C bond cleavage and retention is of great significance for guiding catalyst design and achieving highly selective alcohol oxidation. The following sections will discuss the C–C bond cleavage pathways and C–C bond retention pathways in detail.

### 2.2 Mechanisms of C–C bond cleavage pathways

C–C bond cleavage is a side reaction that must be avoided when using alcohol electrooxidation to obtain high-value products. Its main mechanisms include decarbonylation, base-induced cleavage, and oxidative cleavage. These pathways shorten the carbon chain, lowering the value of the target products. Moreover, they often generate carbon monoxide as an intermediate, which poisons the catalyst surface and severely compromises catalytic durability.

#### 2.2.1 Decarbonylation pathway

Decarbonylation is the most common C–C bond cleavage mechanism on noble metals and occurs mainly on aldehyde intermediates.^46,47^ The aldehyde binds to two adjacent noble metal atoms in a specific adsorption mode, forming a dual-site bond that significantly weakens the C–C bond and promotes its cleavage. Back-donation of electrons from the metal surface to the antibonding orbital of the C–C bond further weakens the bond, ultimately leading to C–C bond cleavage and generating hydrocarbon fragments and carbon monoxide.^48,49^ The hydrocarbon fragments are further oxidized to carbon dioxide or small molecule acids, while carbon monoxide strongly adsorbs on the noble metal surface, causing catalyst poisoning. Especially for Pt, the adsorption energy is extremely high, occupying active sites and leading to catalyst deactivation. Carbon monoxide oxidation requires high potential and surface hydroxyl species; if the oxidation rate of carbon monoxide is lower than its generation rate, the catalyst will gradually deactivate, and the reaction current will decay.

The decarbonylation pathway is closely related to the catalyst structure. This reaction requires multi-site synergy, typically involving two to three adjacent noble metal atoms to simultaneously stabilize the carbon and oxygen of the aldehyde group and the carbon of the hydrocarbon fragment. Different crystal planes exhibit different decarbonylation activities; specific crystal planes of Pt have lower decarbonylation activity, while other planes and step sites show higher activity. Isolated Pt atoms hardly catalyze the decarbonylation reaction.^50^ The coordination number effect is also significant: lower coordination numbers, such as at step sites, enhance C–C bond activation ability.^51^ From a thermodynamic and kinetic perspective, the activation energy of the decarbonylation reaction is usually slightly higher than that of the aldehyde-to-carboxylic acid oxidation pathway.^52^ At low potentials, surface hydroxyl coverage is low, and the decarbonylation pathway may dominate. As the potential increases, surface hydroxyl formation is promoted, accelerating the aldehyde-to-carboxylic acid pathway and thus suppressing decarbonylation.^53^

#### 2.2.2 Base induced C–C bond cleavage

Under strongly alkaline conditions, polyols, especially vicinal diol structures, can undergo C–C bond cleavage through non-electrochemical mechanisms, with this type of cleavage not directly dependent on electrode potential but potentially accelerated by the catalyst surface.^54^ For vicinal diol structures such as EG and glycerol, the mechanism involves deprotonation of hydroxyl groups to form alkoxy species, followed by C–C bond cleavage to generate carbonyl compounds and small-molecule aldehydes or acids, involving carbocation or free radical intermediates and significantly accelerated in alkaline solutions. The key feature of this type of cleavage is that it is a non-electrochemical process that can occur even at open circuit potential and is strongly dependent on hydroxide concentration, with the reaction rate constant increasing approximately tenfold for each unit increase in pH. The catalyst surface can accelerate the process, but it is essentially a coupled homogeneous/heterogeneous reaction in solution.

Compared with the decarbonylation pathway, base-induced cleavage is driven by pH and solution alkalinity rather than electrode potential and surface structure. The intermediates are alkoxy or enolate species rather than adsorbed aldehydes, which not generate carbon monoxide and thus poses no poisoning likelihood. And it has weak potential dependence but strong pH dependence, where the products are similarly low-value small molecules such as formic acid and formaldehyde.

#### 2.2.3 Oxidative cleavage

Under high potential conditions, the catalyst surface forms high-oxidation-state species that can directly attack C–C bond, leading to cleavage.^55,56^ This type of cleavage involves a large number of electron transfers, with products mainly being carbon dioxide and small-molecule acids such as formic acid and acetic acid, which have extremely low value. It typically occurs on the catalyst surface rather than in solution. This process is often accompanied by surface reconstruction and oxidation of the catalyst, further reducing catalytic activity. Oxidative cleavage occurs in the high potential region, where the C–C bond retention pathway has already been suppressed. Therefore, controlling the potential within a suitable window is key to avoiding oxidative cleavage.

### 2.3 Mechanisms of C–C bond retention pathways

When the C–C bond of the alcohol remains intact during electrooxidation, the products can include aldehydes, ketones, or carboxylic acids. For primary alcohol oxidation, the aldehyde is a key intermediate, and its fate determines whether the C–C bond stays intact; this is regulated by three key factors, as discussed in the first three sections below. Among the various products, carboxylic acids are typically the most common due to their stability. Although aldehydes are not particularly stable, several studies still attempt to prepare them, which is the focus of the final section.

#### 2.3.1 Aldehyde desorption rate

The desorption of aldehyde intermediates from the catalyst surface into solution is the most direct pathway for C–C bond retention.^57^ If the aldehyde desorbs rapidly, it is preserved as an aldehyde product with an intact carbon chain. Conversely, if it remains on the surface for a prolonged period, it tends to undergo further reactions, increasing the likelihood of C–C bond cleavage. The desorption rate is influenced by three factors: the molecular structure of the aldehyde, the surface properties of the catalyst, and the applied potential. Aromatic aldehydes, owing to their conjugation stability, exhibit fast desorption rates, while aliphatic aldehydes desorb slowly.^58^ Hydrophobic surfaces promote the desorption of organic species, while hydrophilic surfaces increase their residence time.^59^

#### 2.3.2 Surface hydroxyl coverage

Surface hydroxyl coverage determines the rate of the aldehyde-to-carboxylic acid pathway.^60,61^ High hydroxyl coverage promotes nucleophilic attack, accelerating the conversion of aldehydes to carboxylic acids.^62^ The nucleophilic attack is hindered under low hydroxyl coverage, and aldehydes may either desorb and be retained or undergo decarbonylation cleavage. Hydroxyl coverage is mainly regulated by potential and pH. As potential increases, hydroxyl coverage increases, but excessively high potentials lead to oxide formation, which instead inhibits the reaction. Under alkaline conditions, hydroxyl coverage is higher. Additionally, catalyst composition affects hydroxyl coverage and certain metals such as Au and Bi can adsorb hydroxyl groups at low potentials, providing nucleophilic species.^63,64^

#### 2.3.3 Applied potential

The applied potential is the most direct parameter regulating the fate of aldehyde intermediates. In the low potential region, the metallic surface with low hydroxyl coverage favors aldehyde desorption, leading to high C–C bond retention selectivity and aldehyde as the primary product. In the medium potential region, moderate hydroxyl coverage favors aldehyde oxidation to carboxylic acids, yielding medium-to-high selectivity with carboxylic acids as the primary products. In the high potential region, the formation of surface oxides renders aldehydes susceptible to cleavage, leading to low selectivity and the predominance of C–C cleavage products.^53,65^

From the above discussion, it is evident that C–C bond cleavage pathways entail significant drawbacks. The decarbonylation pathway leads to carbon monoxide poisoning and catalyst deactivation. The base-induced cleavage occurs under strongly alkaline conditions, generating low-value products. The oxidative cleavage occurs at high potentials, producing carbon dioxide and wasting energy. Collectively, these pathways lead to shortened carbon chains, reduced product value, increased energy consumption, and diminished catalyst stability. In contrast, the C–C bond retention pathway generates high-value chemicals such as aldehydes, ketones, and carboxylic acids, involving fewer electron transfers and lower energy consumption, while avoiding carbon monoxide poisoning and achieving better catalyst stability. To achieve high-value conversion, the above mechanistic analysis reveals that the competition between C–C bond retention and cleavage is regulated by multiple factors, including the desorption rate of aldehyde intermediates, surface hydroxyl coverage, applied potential, and substrate molecular structure, among which the atomic arrangement and electronic structure of the catalyst surface are the most critical. The decarbonylation pathway requires multi-site synergy involving adjacent noble metal atoms, while the nucleophilic attack pathway can occur at isolated sites. Therefore, disrupting multi-site synergy and optimizing intermediate adsorption energy through catalyst design represents the strategy for suppressing C–C bond cleavage and realizing highly selective C–C bond retention.

#### 2.3.4 Products from the C–C retaining

The C–C bond-retaining pathway yields different products depending on the oxidation depth. Deep oxidation leads to relatively stable carboxylic acids, which are the most common products. As partially oxidized products, aldehydes are high-value and have also attracted attention as target products.^66–68^ Wang *et al.* found that under alkaline conditions, aldehydes are prone to further oxidation to carboxylic acids *via* nucleophilic attack by OH^−^.^71^ In contrast, under acidic conditions (pH < 6.8), aldehyde conversion is almost completely inhibited, indicating that electrolyte pH plays a crucial role in determining aldehyde selectivity. Besides, Shi *et al.* discovered that under neutral conditions, the Pt/CC catalyst exhibits a distinct current response for ethylene glycol electrooxidation (EGOR) and selectively generates a glyoxal dimer, achieving a faradaic efficiency (FE) of nearly 100% and a selectivity as high as 98.9%.^72^ And the onset potential of EGOR for this catalyst is as low as 0.4 V (*vs.* RHE), the lowest value reported to date. Furthermore, this catalyst demonstrates excellent stability and electrooxidation activity toward various lower alcohols, efficiently converting methanol, ethanol, *n*-propanol, and 1-butanol into their corresponding aldehydes. In addition to adjusting electrolyte pH, Wang *et al.* developed a directional salting-out strategy that successfully achieves highly selective oxidation of alcohols to aldehydes in an alkaline electrocatalytic system, with selectivity reaching 100%.^34^ This strategy, on one hand, effectively reduces the alkalinity of the electrolyte to slow down the oxidation rate of aldehydes. On the other hand, it increases the cation concentration, weakening hydrogen bonding interactions between organic molecules and water, thereby promoting the enrichment of organic molecules at the electrode–electrolyte interface and inhibiting the hydration reaction of aldehydes. These reports indicate that selective oxidation of alcohols to aldehydes relies critically on controlling the electrolyte pH and suppressing aldehyde hydration to prevent further oxidation.

## 3 Catalyst design for C–C bond retain

Currently, catalysts capable of retaining the C–C bond are almost exclusively noble-metal-based ones, which is why we mainly focus on them in this review. Here, we briefly explain why non-precious metal materials tend to favor C–C bond cleavage rather than retention. In particular, Ni-, Co-, and Fe-based non-precious metal materials exhibit good activity toward water electrolysis, organic electro-oxidation, and fuel cell applications in alkaline media.^73–75^ Under an applied electric field, their surfaces undergo self-oxidation, forming high-valent metal oxides/hydroxides (*e.g.*, MO, MOOH) *via* activation of lattice oxygen or surface hydroxyl groups. These *in situ* generated intermediates act as active centers and electron transfer mediators, facilitating the oxidation of organic substrates while being reduced themselves. Consequently, the alcohols are oxidized over the catalyst surface by the metal oxides/hydroxides formed *in situ* on the electrode.^76,77^ As a result, non-noble metal catalysts are constrained by the potential required for active species generation and typically necessitates operation at higher potentials. Such excessively high oxidation potentials not only increase energy consumption but also promote undesired deep oxidation, leading to C–C bond cleavage. This yields oxidation products, as exemplified by ethylene glycol (EG) oxidation, that predominantly consist of low-value formic acid (FA), often accompanied by CO_2_ and other peroxides.^78,79^

In contrast, noble metal catalysts possess the intrinsic ability to directly activate C–H bonds, enabling high selectivity toward C–C bond retention. Numerous studies have demonstrated that noble metals such as Pt, Pd, Au, and Rh exhibit good catalytic activity in the electrooxidation of alcohol molecules.^42,80–83^ Based on the analysis of the reaction mechanism, the selectivity between C–C bond retention and cleavage essentially arises from the interplay between the structure of active sites on the catalyst surface and the adsorption behavior of reaction intermediates. Specifically, the decarbonylation cleavage pathway requires adjacent noble metal atoms to form multi-site synergy to stabilize aldehyde intermediates, whereas the C–C bond retention pathway relies on the rapid desorption of aldehyde intermediates or nucleophilic attack promoted by surface hydroxyl species, which can occur efficiently at isolated sites or bifunctional interfaces. Therefore, the strategy for catalyst design lies in precisely controlling the geometric configuration and electronic structure of active sites to disrupt the multi-site synergy required for decarbonylation while optimizing the kinetics of C–H bond activation and nucleophilic attack. This chapter will start by examining the types of catalysts, analyzing their intrinsic activity and selectivity characteristics, and then systematically summarize the catalyst design strategies reported in the literature, revealing the design principles and mechanisms for achieving high-value conversion through C–C bond retention during alcohol oxidation.

### 3.1 Noble metal catalysts

#### 3.1.1 Pt-based catalysts

**Fig. 3:**
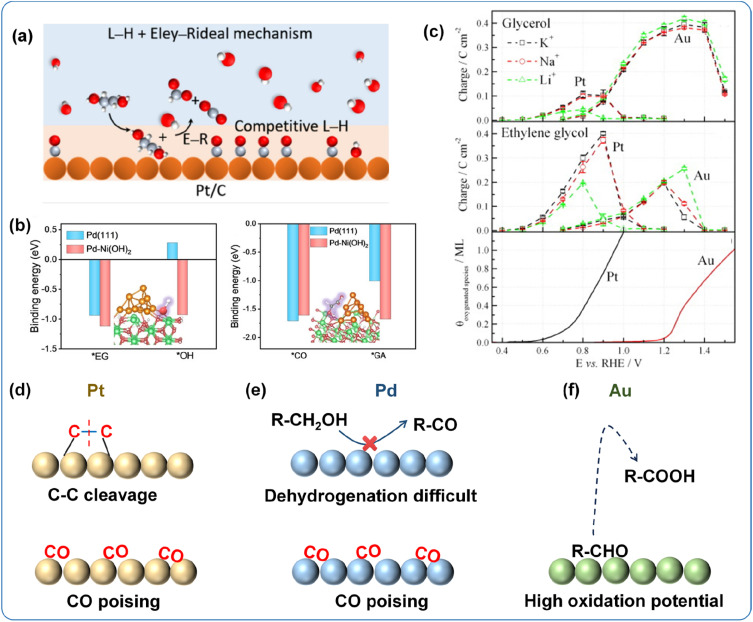
(a) Pt/C catalytic EGOR mechanism;^94^ Copyright 2025, American Chemical Society. (b) Adsorption energies of EG, OH, GA and CO on the surfaces of Pd-based catalyst;^95^ Copyright 2023, Wiley-VCH GmbH. (c) Comparison of oxidation potentials of Pt and Au.^69^ Copyright 2013, Elsevier B.V. Problems that occur when conducting alcohol oxidation on (d) Pt, (e) Pd, and (f) Au catalysts.

Pt has been widely recognized as the most active electrocatalyst for alcohol oxidation, attributed to its unique electronic structure and lower d-band center, which favor the deprotonation of alcohols (Fig. 3a).^84^ However, Pt surfaces are highly prone to decarbonylation reactions, leading to undesired C–C bond cleavage. On the other hand, the CO generated from decarbonylation has an extremely strong adsorption on Pt, causing severe catalyst poisoning and deactivation (Fig. 3d).^85^ To address these issues, the design of Pt-based catalysts is primarily focused on suppressing the decarbonylation pathway and enhancing resistance to CO poisoning.

#### 3.1.2 Pd-based catalysts

Despite the excellent alcohol oxidation performance of Pt, its scarcity, high cost, and high susceptibility to CO poisoning limit its practical large-scale application. Pd, as a fellow Group VIII element, shares similar chemical properties with Pt but offers the advantages of higher abundance and better resistance to CO poisoning, positioning it as a promising alternative to Pt-based catalysts.^38^ However, Pd exhibits a weaker deprotonation ability due to its higher d-band center compared to Pt. Consequently, the cleavage of C–C bonds in alcohols on Pd sites is more difficult, and alcohol oxidation in most Pd-based catalysts predominantly proceeds *via* the C–C bond retention pathway (Fig. 3b).^86^ Although Pd demonstrates better resistance to poisoning, the low surface hydroxyl coverage on monometallic Pd, combined with sluggish reaction kinetics, continues to cause issues associated with carbonyl intermediates and CO poisoning (Fig. 3e).

#### 3.1.3 Au-based catalysts

The catalytic properties of Au are governed by its unique electronic configuration. The fully occupied 5d orbitals effectively shield the nuclear charge, rendering the 6s electrons difficult to remove. As a result, Au requires a higher energy input to undergo oxidation reactions compared to Pt and Pd. Under electrochemical conditions, the oxidation potential of Au is 0.4–0.5 V higher than that of Pt, indicating that a higher potential is required to catalyze alcohol oxidation (Fig. 3c and f).^87^ The excellent chemical stability of Au not only mitigates the likelihood of CO poisoning and carbonyl intermediates but also facilitates the generation of surface hydroxyl species.^70^ Furthermore, adsorbed CO on Au surfaces has been demonstrated to significantly enhance hydroxyl adsorption at low potentials, thereby promoting C–H bond cleavage and boosting the alcohol oxidation activity of Au-based catalysts.^82,89,90^ Thus, although Au requires higher potentials to catalyze alcohol oxidation, its superior chemical stability and favorable interaction with CO offer promising pathways for the design of efficient and durable alcohol oxidation catalysts.

#### 3.1.4 Other noble metal catalysts

Beyond Pt, Pd, and Au, other noble metals such as Rh, Ru, and Ir have also been employed in alcohol electrooxidation.^48^ Rh possesses excellent C–C bond activation capability but is highly prone to C–C bond cleavage during alcohol oxidation, resulting in poor selectivity, which often alloyed with other metals to suppress its cleavage selectivity.^91^ Ru exhibits good dehydrogenation activity and moderate resistance to CO poisoning, but it is susceptible to the formation of high-valence oxides at high potentials, which causes deactivation.^92^ Ir is recognized for its excellent stability but has a strong affinity for CO adsorption renders it susceptible to poisoning.^93^

### 3.2 Catalyst design strategies

#### 3.2.1 Single-atom catalysts

**Fig. 4:**
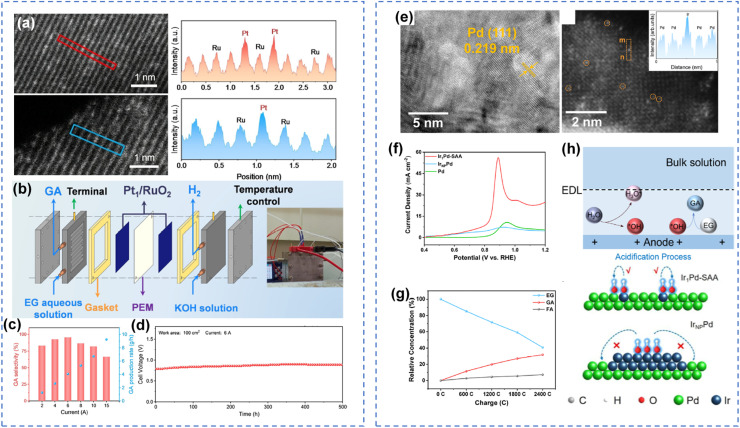
Single-atom catalytic oxidation of alcohols. (a) HAADF-STEM images and the intensity, (b) schematic illustration of a two-electrode MEA flow electrolyzer, (c) GA selectivity and yield rate, (d) stability test in flow electrolyzer of Pt_1_/RuO_2_;^96^ Copyright 2025, Wiley-VCH GmbH. (e) HRTEM image and HADDF-STEM images, (f) LSV curves, (g) the concentrations of EG, GA, and FA during 2400C charge input, (h) schematic diagram of surface-localized microenvironmental acidification and *OH spillover behavior on Ir_1_Pd-SAA;^97^ Copyright 2026, American Chemical Society.

Single-atom catalysts (SACs), which disperse noble metals as isolated atoms on supports or host metal surfaces, achieve atomic-scale site isolation and represent the ultimate strategy for suppressing the decarbonylation pathway. By existing as isolated atoms, noble metals completely disrupt the multi-site synergy necessary for decarbonylation, thereby fundamentally inhibiting C–C bond cleavage. Chen *et al.* incorporated single-atom Pt into RuO_2_ (Pt_1_/RuO_2_) *via* a simple wet impregnation method, achieving efficient and stable conversion of EG to glycolic acid under alkaline conditions (Fig. 4a).^94^ Electrocatalytic tests showed that Pt_1_/RuO_2_ exhibited excellent mass activity (8.09 A mg_Pt_^−1^), as well as exceptionally high glycolic acid faradaic efficiency (FE = 95.3%) and selectivity (96.9%). The single-atom Pt precisely controlled the single-site adsorption configuration of EG, suppressing C–C bond cleavage, while RuO_2_ enhanced surface hydroxyl coverage, effectively reducing the adsorption of CO intermediates and preventing catalyst poisoning. The catalysts exhibited stable operation for 500 h under industrial-scale conditions, achieving a glycolic acid production rate of 4.06 g h^−1^ (Fig. 4b–d). Furthermore, they constructed an Ir_1_Pd single-atom alloy catalyst to facilitate water dissociation in neutral media. The hydroxyl species generated at Ir single sites are directionally transferred to adjacent Pd sites *via* the Ir–Pd atomic-scale interface, where they actively participate in EG oxidation. This design achieves multi-scale synergistic regulation encompassing spatial, temporal, and electronic effects (Fig. 4e).^95^ The Ir_1_Pd catalyst achieved a glycolic acid selectivity of 80.1%, a FE of 76.3%, and stability exceeding 330 h, thereby providing a new approach for the design of alcohol oxidation catalysts in neutral media (Fig. 4f–h). Based on this concept, Duan *et al.* developed a nickel oxide-supported single-atom Ru catalyst (Ru_1_-NiO) capable of electrocatalytically oxidizing biomass-derived alcohols to aldehyde products under neutral conditions.^69^ The single-atom Ru significantly enhanced the activity of 5-hydroxymethylfurfural oxidation by promoting water dissociation. Furthermore, this Ru_1_-NiO catalyst could be applied to the electrooxidation of various biomass-derived alcohols to aldehydes, providing a direction for the rational design of highly selective alcohol oxidation electrocatalysts in neutral media.

**Fig. 5:**
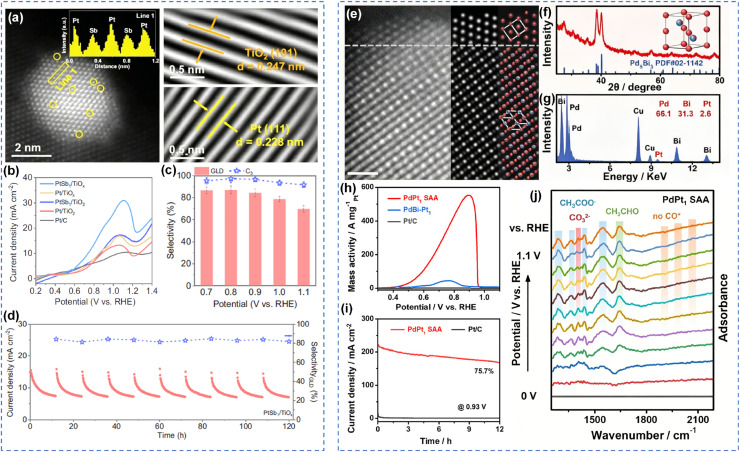
Single-atom alloy catalytic oxidation of alcohols. (a) HAADF-STEM images and corresponding IFFT images, (b) GOR polarization curves, (c) selectivity of GLD and C_3_ products at various potentials (d) chronoamperometry profiles and GLD selectivity on PtSb_1_/TiO_*x*_ for ten cycles;^79^ Copyright 2025, Wiley-VCH GmbH. (e) Aberration-corrected HAADF-STEM image, (f) XRD pattern, (g) EDX spectrum; (h) CVs, (i) long-term CA curve, (j) *in situ* FTIR spectrum of PtAu_1_ SAA.^80^ Copyright 2026, Wiley-VCH GmbH.

Beyond conventional single-metal systems, single-atom alloys synergistically combine the advantages of single-atom catalysts and nanoalloys, positioning them as ideal model systems for electrocatalytic studies. Wang *et al.* developed a PtSb single-atom alloy catalyst supported on non-stoichiometric TiO_2_ (PtSb_1_/TiO_*x*_), which exhibited excellent glycerol oxidation performance in neutral conditions (Fig. 5a).^96^ The catalyst achieved 87% glyceraldehyde selectivity and 97.2% total C_3_ product selectivity, with stability exceeding 120 h (Fig. 5b–d). The PtSb_1_ single-atom alloy enhanced glyceraldehyde desorption, thereby improving C–C bond retain selectivity. Meanwhile, the atomically dispersed Sb stabilized Pt sites through Pt–Sb bonds, mitigating oxidative deactivation during long-term glycerol oxidation. In a membrane electrode assembly electrolyzer, the system achieved simultaneous production of C_3_ products and H_2_O_2_. Luo *et al.* designed a tensile-strained PdPt_1_ single-atom alloy surface by anchoring single Pt atoms on the surface of Pd_2_Bi_3_ core-ultrathin Pd shell octahedral intermetallic compounds (Fig. 5e–g).^97^ This unique PdPt_1_ single-atom alloy surface exhibited record-high mass activity (34.73 A mg_Pt+Pd_^−1^) and excellent durability for ethanol oxidation in alkaline electrolyte, outperforming state-of-the-art electrocatalysts (Fig. 5h–j). Density functional theory calculations revealed that the tensile strain and the introduction of single Pt atoms optimized the electronic structure of the catalyst, enhanced ethanol adsorption and electron transfer, lowered the reaction energy barrier, and thus significantly improved EOR performance.

#### 3.2.2 Monometallic materials

**Fig. 6:**
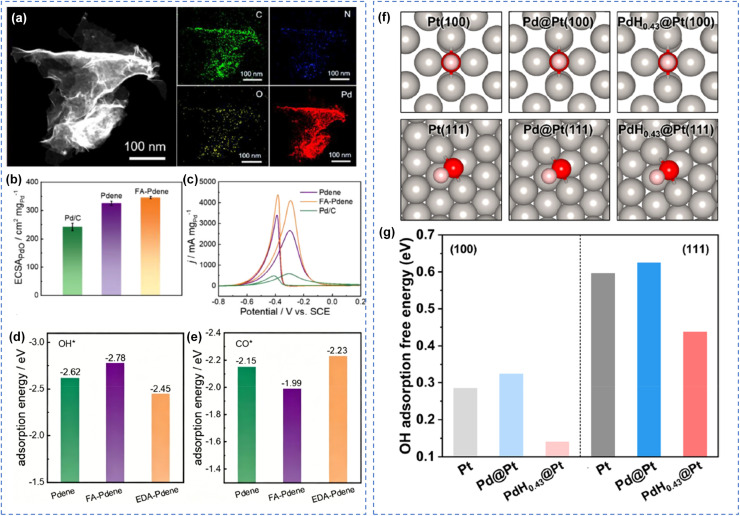
Monometallic metal materials catalytic oxidation of alcohols. (a) Dark-field TEM image and the associated EDX elemental mappings, (b) ECSAs derived from PdO reduction peaks in CVs, (c) CVs of the catalysts in 1.0 M KOH + 1.0 M ethanol solution, the calculated (d) OH*, (e) CO adsorption energy on Pdene, FA-Pdene, and EDA-Pdene surfaces.^98^ Copyright 2025, Wiley-VCH GmbH. (f) Adsorption configurations and (g) adsorption free energies of OH on the surfaces of Pt with different faces.^99^ Copyright 2021, American Chemical Society.

Pure metal materials represent the earliest class of alcohol oxidation catalysts. Pt, Pd, and Au each display unique catalytic behaviors. Their performance is significantly governed by crystal plane structure, morphology, and particle size. Although morphology control can expose specific crystal planes and optimize catalytic performance to a certain degree, pure metal materials generally struggle to achieve a satisfactory balance between activity and selectivity. The intrinsic properties of single metals impose inherent limitations on further performance enhancement, thereby necessitating modification through advanced strategies such as alloying and compositing. Li *et al.* developed an efficient catalyst system based on alcohol amine-modified Pd metallene (FA-Pdene). They demonstrated that FA molecules not only enhance the accessibility of active sites but also significantly improve the catalytic performance of Pdene in alcohol oxidation through electronic modulation effects (Fig. 6a).^98^ Specifically, FA promotes dispersion, thereby increasing the electrochemical active surface area. Concurrently, the electron-withdrawing nature of FA induces an electron-deficient state on the Pdene surface, which promotes the adsorption of electron-rich intermediates such as OH* and facilitates the desorption of electron-deficient toxic intermediates such as CO*. In the ethanol oxidation reaction, FA-Pdene exhibited a 54.5% increase in mass activity and a 46.3% increase in specific activity relative to unmodified Pdene (Fig. 6b–e). Additionally, the catalyst demonstrated excellent operational stability, superior resistance to CO poisoning, and enhanced complete oxidation pathway selectivity. This fullerene-mediated catalytic effect offers new avenues for advancing the performance of metal-based catalytic systems.

The intrinsic limitations of single metals necessitate advanced modification strategies. Moreover, even for a given metal, catalytic activity and selectivity are facet-dependent, since variations in atomic distribution and packing density across crystal planes give rise to distinct electronic effects. Thus, both compositional and structural engineering are essential for optimizing catalyst performance. Zhang *et al.* converted the Pd core to PdH_0.43_ through hydrogen intercalation. The lattice expansion of the Pd core induced lattice expansion of the Pt shell, significantly promoting alcohol oxidation reactions on Pt(100) and Pt(111) facets.^99^ The resulting catalyst with exposed Pt(111) facets exhibited a Pt mass-specific activity of 14.86 A mg Pt^−1^ for ethanol oxidation, 25.19 times higher than that of commercial Pt/C. Through lattice expansion, the catalytic performance on both Pt(100) and Pt(111) facets was significantly improved, attributed to enhanced OH adsorption. This work not only paves the way for lattice engineering of specific crystal facets in nanomaterials to enhance electrocatalytic activity but also provides a promising strategy for the rational design and preparation of highly efficient catalysts (Fig. 6f and g).

#### 3.2.3 Alloy catalysts

Alloy catalysts, which combine noble metals with secondary or tertiary metals to form solid solutions, intermetallic compounds, or core–shell structures, achieve a synergistic interplay between geometric dilution and electronic regulation, positioning them as the most promising material systems for practical applications. Sun *et al.* synthesized surface Ga-modified PtGa nanoalloy particles. The Ga-(H_2_O)_*x*_ species formed by Ga atoms adsorbing water molecules effectively isolated continuous Pt sites. *In situ* electrochemical infrared spectroscopy revealed that ethanol molecules did not undergo C–C bond cleavage after adsorption on the catalyst surface, with intermediates remaining as C_2_ species (acetaldehyde), thus avoiding the C_1_ (CO) poisoning pathway.^100^ As the potential increased, Ga-(H_2_O)_*x*_ released protons to form Ga-(OH)_*x*_, and these OH species promoted further oxidation of acetaldehyde, enabling complete oxidation of acetaldehyde to CO_2_ at low to medium potentials (0.3–0.55 V *vs.* SCE). This study proposed a new mechanism for ethanol oxidation, which provides a good idea for the C–C bond retention.

**Fig. 7:**
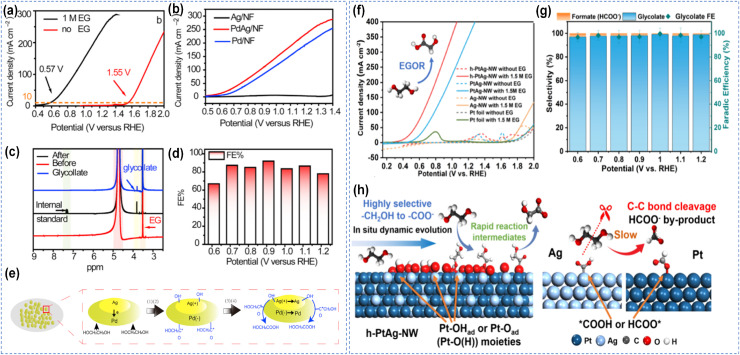
Binary alloy catalytic oxidation of alcohols. (a and b) LSV curves of catalysts in 0.5 M KOH with and without addition of 1 M ethylene glycol, (c) ^1^H NMR spectra of the electrolyte before and after 2 h anodic ethylene glycol oxidation on a PdAg/NF electrode, (d) FEs of PdAg/NF for glycolic-acid production at varied voltages, (e) the probable mechanism of the synergetic catalytic effect for ethylene glycol oxidation on the PdAg/NF catalyst;^101^ Copyright 2021, Elsevier B.V. (f) LSV profiles with or without 1.5 M EG, (g) selectivity and FE for glycolate and formate byproduct with different potentials, (h) schematic drawing of the reaction pathways, the influence of surficial oxidation and Pt–O(H) moieties on h-PtAg-NW, and the C–C dissociation on the Pt or Ag surface.^84^ Copyright 2025, American Chemical Society.

Binary alloys represent the most extensively studied systems. Shi *et al.* prepared PdAg alloy nanomaterials.^101^ The oxidation potential was substantially lower than that of the OER, while the current density was considerably higher (Fig. 7a and b). Product analysis confirmed successful C–C bond retention, with glycolic acid as the major product, achieving a FE as high as 97% (Fig. 7c and d). This enhanced performance is primarily attributed to the optimized adsorption of OH* species on the catalyst surface upon Ag incorporation, which facilitates the rapid oxidation of CH_2_OH–CO* and CO intermediates (Fig. 7e). Li *et al.* synthesized hollow-structured bimetallic PtAg nanowires (h-PtAg-NW) using an *in situ* dynamic evolution strategy for the electrooxidation of EG to glycolic acid.^84^ The resulting PtAg nanostructure effectively suppresses C–C bond cleavage. The current density of 350 mA cm^−2^ is achieved at an applied potential of 1.0 V, with a FE for C–C retain pathway approaching 97% (Fig. 7f and g). The formation of Pt–O(H)_ads_ species on the catalyst surface modulates the surface electronic structure, promotes the selective adsorption of EG *via* its single-side hydroxyl groups, and consequently enhances the EGOR performance of Pt (Fig. 7h). Similar to PtAg, PtBi intermetallic compounds compounds operate *via* a bifunctional mechanism, in which Bi preferentially adsorbs OH* at low potentials and transfers active oxygen species to adjacent Pt sites, thereby accelerating the oxidation of aldehyde intermediates and effectively suppressing their decarbonylation cleavage.^85^ Besides, various transition metals and oxides/hydroxides (such as Ni, Co, Mn, Cu) have also been used to promote OH* adsorption during alcohol oxidation, exhibiting superior alcohol oxidation activity and stability.^35,102,103^

**Fig. 8:**
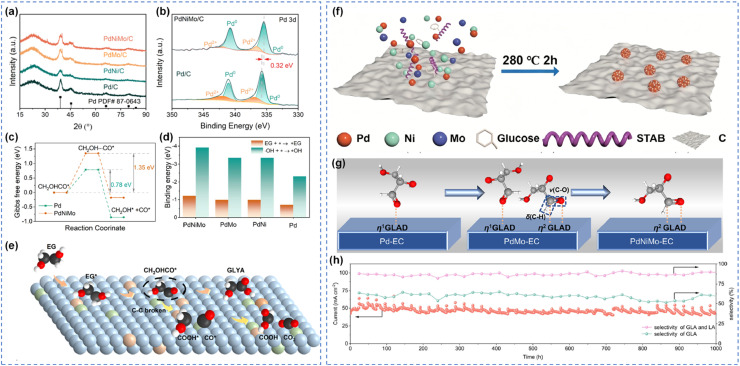
Multi-component alloys catalytic oxidation of alcohols. (a) XRD spectra of different Pd-based catalysts, (b) XPS spectra of Pd 3d, (c) Gibbs free energy during C–C bond cleavage in CH_2_OH–CO* on Pd and PdNiMo surfaces, (d) adsorption energies of EG and *OH, (e) reaction pathways for C–C bond breaking in the EGOR process of PdNiMo/C;^104^ Copyright 2025, the Royal Society of Chemistry. (f) Schematic of the synthesis procedure, (g) catalytic mechanism investigation, (h) long-term GEOR test for MEA flow electrolyzer by using interval CA strategies.^60^ Copyright 2026, Wiley-VCH GmbH.

**Fig. 9:**
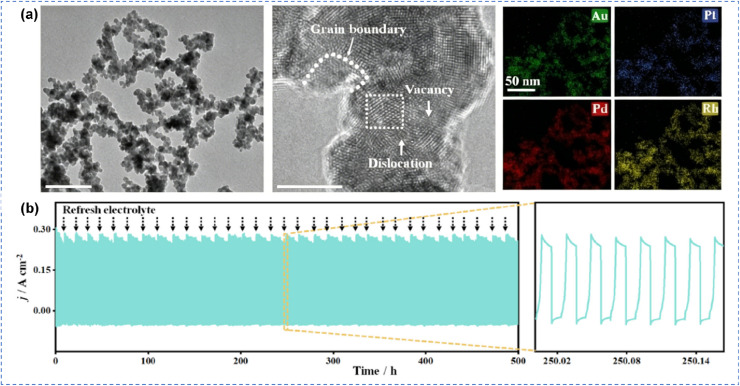
Multi-component alloys catalytic oxidation of alcohols. (a) TEM, HR-TEM and HAADF-STEM and the corresponding EDS mapping images of AuPtPdRh MEA, (b) stability testing of AuPtPdRh MEA ‖ Pt/C two-electrode MFE system under PS-SHE-0.25 mode for 500 h.^105^ Copyright 2026, Wiley-VCH GmbH.

Multi-component alloys, containing three or more metals, offer more precise regulation of catalytic performance. In ternary alloys, each metal plays a distinct yet synergistic role, collectively enabling high-performance alcohol oxidation. Wei *et al.* co-doped the oxophilic transition metals Ni and Mo into a Pd/C catalyst to enhance the EG oxidation performance (Fig. 8a and b).^104^ Compared to Pd/C, the mass activity increased by 1.86 times, and the operational stability was extended fivefold. The introduction of Ni and Mo promoted the adsorption of EG and OH* on the electrode surface, thereby accelerating electron transfer between EG and the catalysts, effectively lowering the reaction energy barrier and enhancing oxidation kinetics. Mechanistically, Ni increased the energy barrier for C–C bond cleavage of the CH_2_OH–CO* intermediate, activated the C=O bond, and promoted its subsequent oxidation to glycolic acid. Consequently, the formation of C_1_ byproducts was effectively suppressed, and reaction stability was significantly improved (Fig. 8c–e). Chen *et al.* reported a ternary PdNiMo alloy catalyst that achieves efficient and stable conversion of glycerol to glycerate through synergistic electronic structure modulation and surface OH coverage optimization (Fig. 8f).^60^ Specifically, Mo incorporation downshifts the d-band center and weakens OH* adsorption, whereas Ni introduction optimizes the balance between OH*-covered sites and free Pd sites. Consequently, the resulting PdNiMo catalyst exhibites excellent glycerol oxidation performance, achieving a high current density of 171 mA cm^−2^ at 0.8 V and glycerate selectivity of 67.5%. In addition, the catalyst demonstrated outstanding durability, maintaining a current density exceeding 50 mA cm^−2^ at a cell voltage of 1.2 V for over 1000 h (Fig. 8g and h). This work elucidates the critical role of surface OH* coverage in selective oxidation and provides design principles for advanced electrocatalysts for renewable energy-driven electrosynthesis. Furthermore, Deng *et al.* designed an AuPtPdRh medium-entropy alloy aerogel featuring multi-site synergy, which was integrated with a novel potential scanning-step hybrid electrolysis dynamic modulation strategy (Fig. 9a).^105^ The multi-site potential relay catalytic mechanism reduced the overall energy barrier, while the progressive pre-enrichment-pulse cleaning regeneration cycle enhanced catalytic activity and stability. The system achieved FE of 98% for glyceraldehyde and yield of 8.82 mmol cm^−2^ h^−1^ in a membrane-free flow cell, operating stably for 500 h at ampere-level current densities (Fig. 9b). Notably, this method also demonstrated universality for the electrooxidation of methanol, ethanol, and propanol, providing a new platform for the valorization of biomass-derived alcohols.

**Fig. 10:**
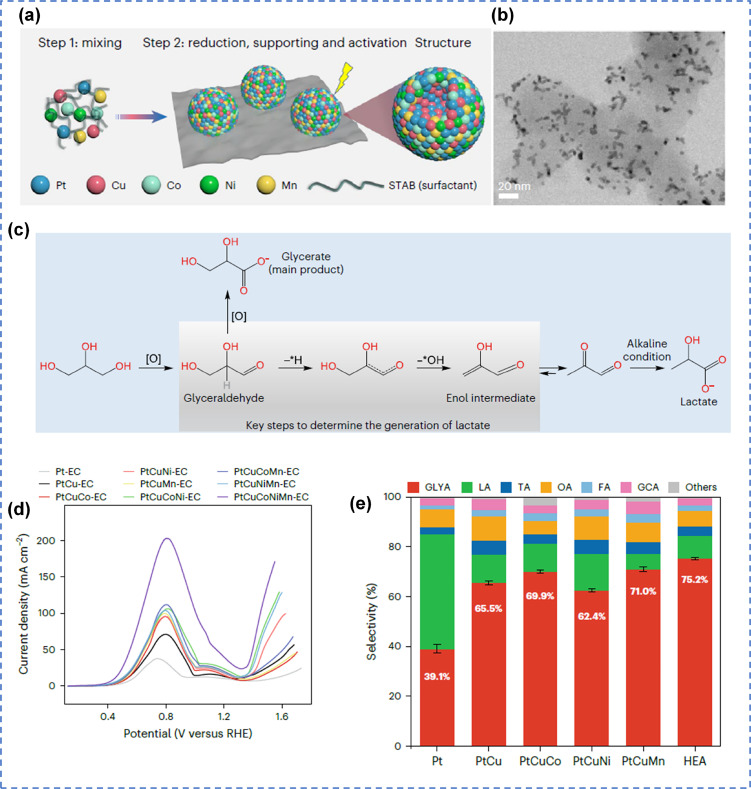
High-entropy alloys catalytic oxidation of alcohols. (a) Schematic of the synthesis procedure, (b) TEM image, (c) proposed pathways to glycerate and lactate, (d) LSV curves of GEOR over different nanocatalysts, (e) products distributions at 0.8 V over different catalysts.^106^ Copyright 2025, Springer Nature.

In recent years, high-entropy alloys composed of five or more elements have attracted extensive research interest. Compared with traditional tri-metallic or tetra-metallic alloys, high-entropy possess distinctive physicochemical properties, including pronounced lattice strain and distortion, tunable electronic structures, and strong synergistic effects, often collectively referred to as the “cocktail effect”. Leveraging these remarkable characteristics, researchers hypothesized that Pt-based high-entropy alloys could address the issues of low activity and selectivity in the conversion of glycerol to glycerate. Chen *et al.* synthesized nanocatalyst featuring a PtCu-rich core and a high-entropy alloy surface (PtCuCoNiMn-EC) using a wet chemical method coupled with electrochemical surface reconstruction (Fig. 10a and b).^106^ The prepared PtCuCoNiMn-EC exhibited extremely high glycerol electrooxidation activity at 0.8 V (5.4 times higher than Pt/C) and excellent glycerate selectivity (75.2%), marking one of the best performances reported to date (Fig. 10c and d). The electrolyzer constructed in this study successfully achieved a current density of approximately 200 mA cm^−2^ and 71.8% glycerate selectivity over 210 h, thereby providing an effective strategy for the selective electrocatalytic cascade oxidation of biomass to produce high-value chemicals.

#### 3.2.4 Composite materials

Composite materials combine noble metals with oxides, hydroxides, carbon materials, and other supports, leveraging interfacial synergistic effects between the support and the noble metal to significantly enhance catalytic performance. The central design principle of such materials is the deliberate construction of metal-support interfaces, which facilitate bifunctional synergy between the two components.

**Fig. 11:**
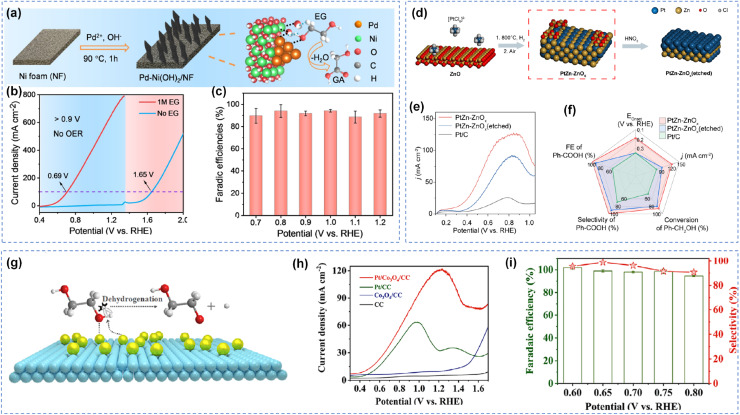
Composite materials catalytic oxidation of alcohols. (a) Schematic illustration of the synthesis of Pd-Ni(OH)_2_ on NF, (b) LSV curves, (c) FE for GA production at designated;^86^ Copyright 2023, Wiley-VCH GmbH. (d) Schematic illustration of the formation of PtZn–ZnO_*x*_ (etched), (e) LSV curves of different electrocatalysts in 1.0 M KOH with 0.1 M Ph–CH_2_OH, (f) comparison of Ph–CH_2_OH oxidation over different catalysts;^107^ Copyright 2025, American Chemical Society. (g) Schematic diagram of direct dehydrogenation mechanism; (h) LSV curves of obtained electrocatalysts for EGOR, (i) FE and selectivity of Pt/Co_3_O_4_/CC.^72^ Copyright 2025, Wiley-VCH GmbH.

In metal-oxide composite systems, reducible oxide supports such as CeO_2_ and TiO_2_ provide abundant hydroxyl species through their oxygen vacancies, promoting nucleophilic attack pathways.^88^ Au-based composite electrode employing CoOOH as supports have been developed for various alcohol oxidation reactions, demonstrating excellent catalytic activity. Specifically, the Au/Ni(OH)_2_ catalyst achieved high current densities while maintaining over 90% selectivity for target products.^37^ Chen *et al.* further prepared Ni-supported Pd catalyst (Pd-Ni(OH)_2_/NF), in which the Ni(OH)_2_ support adsorb OH* at relatively low potentials, thereby promoting C–H and O–H bond activation during EG oxidation and achieving a high current density of 600 mA cm^−2^ at 1.15 V *vs.* RHE (Fig. 11a–c).^86^ Additionally, Chen *et al.* developed a PtZn–ZnO_*x*_ catalyst that enabled efficient benzyl alcohol oxidation at a low potential of 0.725 V, achieving 99.5% selectivity for benzoic acid.^107^ The unsaturated coordination Zn atoms at the PtZn–ZnO_*x*_ interface promote the adsorption of both benzyl alcohol and benzaldehyde, as well as the generation of electrophilic OH* species. Furthermore, the PtZn–Zn_*x*_ interface lowers the energy barrier for the coupling of OH* with benzaldehyde, thereby enhancing catalytic activity and selectivity (Fig. 11d–f). Beyond ZnO supports, Shi *et al.* loaded Pt onto Co_3_O_4_/CC, achieving nearly 100% FE, 99.0% selectivity, and a yield of 204.9 µmol h^−1^ cm^−2^ for glycolaldehyde dimerization in neutral electrolyte.^72^ Combined characterization and theoretical calculations indicate that the Co_3_O_4_ support enhanced the dehydrogenation ability of Pt by increasing its electron density, thereby overcoming the limitations of traditional active oxygen mechanisms with respect to reaction conditions and selectivity (Fig. 11g–i). This work not only provides new perspectives for electrocatalyst design and reaction pathway regulation but also lays theoretical and technical foundations for the green synthesis of chemicals featuring low energy consumption and high atom economy.

**Fig. 12:**
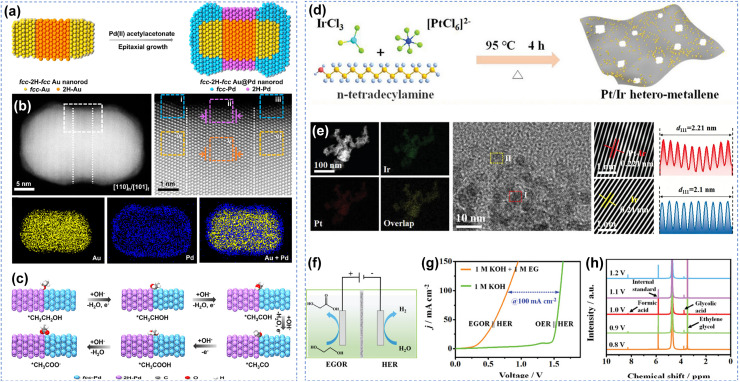
Heterostructure materials catalytic oxidation of alcohols. (a) Schematic illustration for the synthesis of heterophase Au@Pd nanorod, (b) HAADF-STEM image, enlarged HAADF-STEM image, and corresponding EDS elemental mapping and overlapped images, (c) schematic illustration for the ethanol oxidation pathway on the expanded 2H (110)/fcc (101) phase boundary of Pd;^108^ Copyright 2021, American Chemical Society. (d) Schematic diagram, (e) HAADF-STEM and corresponding element mapping, HRTEM images, the lattice pattern and corresponding atomic absorption intensity profile of the selected area for Pt/Ir hetero-metallene, (f) schematic illustration for two-electrode EG-assisted water splitting system, (g) LSV curves, (h) ^1^H NMR spectra of the products of anodic EGOR at different potentials.^80^ Copyright 2024, Wiley-VCH GmbH.

Heterostructures have also attracted extensive attention because their distinct phases can provide different functions. Zhang *et al.* synthesized heterophase Au@Pd core–shell nanorods with a well-defined fcc-2H-fcc structure through wet-chemical epitaxial growth (Fig. 12a and b).^108^ The main pathway for ethanol oxidation on this material was the C_2_ pathway yielding acetate, and it exhibited fast reaction kinetics. Experimental results combined with theoretical calculations indicated that the excellent performance of this material originated from its unconventional 2H phase, unique 2H/fcc phase interfaces, and lattice-expanded Pd shell. This precise phase engineering strategy provides a new method for synthesizing heterophase nanomaterials with well-defined structures and offers an ideal platform for studying structure–performance relationships (Fig. 12c). Wang *et al.* anchored Pt nanoclusters on ultrathin two-dimensional Ir metallene, constructing a Pt/Ir heterostructure (Pt/Ir hetero-metallene) with Pt–Ir interfaces for the conversion of EG to glycolic acid coupled with hydrogen production (Fig. 12d and e).^80^ With the assistance of EG oxidation, the Pt/Ir‖Pt/Ir hetero-metallene two-electrode water electrolysis system achieved a cell voltage as low as 0.36 V at 10 mA cm^−2^. Moreover, the FE for EG to glycolic acid conversion reached 87% (Fig. 12f–h). The excellent performance of this novel heterostructure originated from the charge redistribution and strain effects induced by Pt–Ir interactions at the heterointerface, as well as the larger specific surface area and more active sites provided by the metallene structure.

The design of noble metal catalysts has evolved from discovering intrinsic characteristics to actively controlling them, progressing from single metals to multi-element alloys and from single atoms to composite materials. Among single-metal catalysts, Pt exhibits high activity but poor selectivity, Au demonstrates excellent selectivity but low activity, and Pd falls between the two. Alloy catalysts effectively break the trade-off between activity and selectivity through geometric dilution and electronic regulation, with systems such as PtAg, PtGa, and PdAg demonstrating excellent C–C bond retention performance. Single-atom catalysts fundamentally suppress decarbonylation through atom site isolation, achieving breakthroughs in selectivity. Composite materials further expand the space for performance optimization through metal–support interfacial synergy. The synergistic application of these design strategies provides solid support for achieving highly selective C–C bond retention in alcohol oxidation.

## 4 Techno-economic analysis of alcohol oxidation

In this section, we extend our analysis to evaluate how this C–C bond-retaining electrooxidation of alcohols contributes to energy-saving hydrogen production by replacing of the oxygen evolution reaction (OER). Several quantitative metrics are also estimated to provide guidance for future research.

**Fig. 13:**
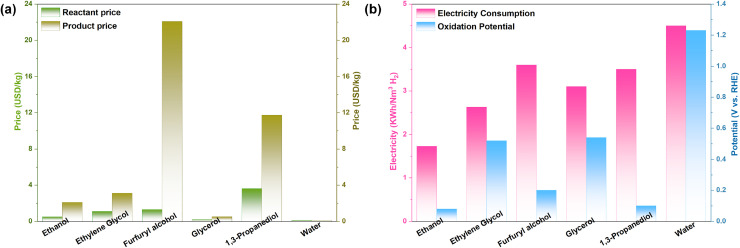
(a) Market prices of alcohols and their oxidation products, and (b) theoretical oxidation potentials and electricity consumption for the C–C bond-retaining electrooxidation of alcohols. The theoretical potentials are calculated for the oxidation of the corresponding alcohols to acetic acid, glycolic acid, lactic acid, 3-hydroxypropionic acid, and furoic acid, respectively.

First, a comparison of the market prices of alcohol reactants and their corresponding oxidation products (Fig. 13a) reveals that, although alcohol substrates are more expensive than water, the resulting products are typically far more valuable than the reactants themselves, offering substantial economic gains.^109^ In contrast to conventional water electrolysis, where the anodic product (oxygen) has almost no commercial value and poses explosion risks when mixed with cathodically generated hydrogen, alcohol oxidation inherently avoids the formation of explosive H_2_/O_2_ mixtures. These advantages highlight the economic and safety merits of coupling alcohol oxidation with hydrogen production.

Furthermore, we compare the theoretical oxidation potentials of various alcohols (Fig. 13b). The theoretical potential for the OER is 1.23 V, which is substantially higher than those of alcohol oxidation reactions (typically below 0.6 V), demonstrating that replacing the OER with alcohol oxidation can significantly reduce electrical energy consumption.^110,111^ We also compare the differences in energy consumption: when the OER is replaced by alcohol oxidation, most studies achieve lower energy consumption (<4 kWh Nm^−3^ H_2_) compared to conventional water splitting (typically 4.5–5 kWh Nm^−3^ H_2_). Nevertheless, there remains a lack of research comparing energy consumption at practically relevant high current densities. Thus, alcohol oxidation offers substantial advantages in both thermodynamic potential and electrical energy efficiency, underscoring its promise for energy-saving hydrogen production.

## 5 Conclusions and outlook

This review systematically summarizes recent progress in noble metal-based electrocatalysts for the selective oxidation of biomass-derived alcohols with C–C bond retention as an alternative to the oxygen evolution reaction, with particular emphasis on reaction mechanisms and catalyst design strategies. At the mechanistic level, the primary challenge lies in the competition between C–C bond retention and cleavage. The retention pathway relies on rapid aldehyde desorption or nucleophilic attack facilitated by surface hydroxyl species, which can efficiently occur at isolated sites or bifunctional interfaces. The applied potential, surface hydroxyl coverage, aldehyde desorption rate, and substrate structure collectively govern the reaction pathway. This mechanistic insight directly guides catalyst design: disrupting multi-site synergy and optimizing intermediate adsorption energy are core strategies for suppressing C–C bond cleavage and achieving highly selective retention. At the catalyst design level, various strategies, including single-atom catalysts, alloys, and composite materials provide robust support for achieving highly selective C–C bond retention in alcohol oxidation. Notably, alcohol oxidation coupled with electrolysis of water for hydrogen production technology achieve 40–50% energy savings compared to conventional water electrolysis, while converting low-cost biomass-derived alcohols into high-value chemicals, thereby significantly enhancing overall process economics. Collectively, these advances lay a solid foundation for transitioning from laboratory research to practical applications.

Based on the above analysis of reaction mechanisms and catalyst design, future research on alcohol oxidation-assisted hydrogen production can be advanced in the following directions.

First, the scope can be expanded from model molecules to real biomass systems. To date, most studies have focused on pure model substrates such as glycerol, ethylene glycol, and benzyl alcohol. However, biomass-derived alcohols inherently contain various impurities, including salts, fatty acids, and lignin residues, which can profoundly affect catalyst performance and system stability by poisoning active sites, obstructing mass transport channels, and degrading membrane structures. To address these challenges, future research should prioritize the development of impurity-tolerant catalyst design strategies. These may include introducing anti-poisoning sites, constructing hydrophobic surfaces to suppress impurity adsorption, and designing self-cleaning reaction interfaces. Simultaneously, systematic investigations under membrane electrode assembly conditions that closely mimic real operating environments are essential to elucidate the effects of impurities on catalyst selectivity and stability. Such efforts will help accelerate the transition of this technology toward practical biomass refining scenarios.

Second, understanding the dynamic reconstruction of catalysts under industrially relevant high current densities (>500 mA cm^−2^) remains a critical yet underexplored challenge. Unlike mild laboratory conditions, high-current operation introduces extreme local environments – steep pH gradients, intense gas bubble formation, and elevated temperatures, which fundamentally alter catalyst reconstruction pathways, accelerating phase transformations and potentially generating unique metastable active species. Future research should prioritize *operando* characterization techniques (*e.g.* Raman, XAS, DEMS) adapted to high-current conditions to track real-time structural evolution and establish structure–performance relationships. Moreover, systematic investigation of how current density modulates reconstruction pathways could enable reconstruction engineering as a strategy to tune selectivity toward desired C–C bond-retaining products. The development of reconstruction-resilient catalysts through defect engineering, heteroatom doping, or support interactions is also essential to prevent over-oxidation and deactivation. Ultimately, bridging the gap between low-current mechanistic studies and high-current practical applications through multi-scale modeling will be crucial for scaling up alcohol oxidation-assisted hydrogen production technologies.

Third, membrane compatibility remains a critical yet often overlooked bottleneck for practical implementation. Current anion exchange membranes (AEMs) are well-suited for the alkaline conditions that favor C–C bond retention, but they suffer from insufficient chemical stability when exposed to organic substrates (*e.g.*, alcohols, aldehydes) and their oxidation products. This leads to membrane swelling, degradation, and increased alcohol crossover. Particularly, this crossover not only reduces faradaic efficiency by consuming oxidants at the cathode but also contaminates the hydrogen product and poses safety risks. Future research should therefore prioritize the development of next-generation AEMs or bipolar membranes with enhanced chemical resistance and reduced permeability to organic molecules. Additionally, innovative membrane electrode assembly designs, such as incorporating protective interlayers or optimizing flow fields to create concentration gradients that minimize crossover, are urgently needed. Addressing these membrane compatibility issues is essential to achieving the long-term stability (>5000 h) and high efficiency required for industrial-scale deployment.

Fourth, product separation represents an indispensable yet often underestimated step in practical implementation. Unlike conventional water electrolysis, where the anodic product (oxygen) is simply vented, alcohol oxidation systems generate high-value organic products (*e.g.*, aldehydes, carboxylic acids) dissolved in the electrolyte, necessitating efficient and cost-effective separation to realize economic viability. Current separation methods, such as liquid–liquid extraction, distillation, and crystallization, are often energy-intensive, especially when dealing with dilute product streams and complex mixtures containing unreacted substrates, byproducts, and electrolytes. Future research should focus on developing *in situ* product separation technologies, such as membrane-based separation (*e.g.*, nanofiltration, electrodialysis, pervaporation), that can continuously remove target products from the reaction environment, thereby preventing overoxidation and reducing downstream purification costs. Additionally, integrating product separation with electrolyzer design through two-phase electrolyte systems or reactive extraction, could simplify the overall process and enhance both economic and environmental sustainability. Ultimately, the development of efficient, low-energy, and scalable separation strategies is critical for translating laboratory-scale successes into commercially viable biomass and plastic valorization technologies.

In summary, by modulating the atomic arrangement and electronic structure of noble metal catalysts, it is possible to effectively suppress C–C bond cleavage while maintaining high dehydrogenation activity, thereby providing a feasible path toward highly selective C–C bond retention in alcohol oxidation. As catalyst design strategies continue to deepen and reactor engineering technologies advance, alcohol oxidation-assisted hydrogen production is expected to play an increasingly pivotal role in future green hydrogen energy systems and biomass refining.

## Author contributions

W. J. Zhang, J. Li, and Z. D. Wei structured this review. B. R. Wang collected papers related to the topic. The manuscript was revised by all authors.

## Conflicts of interest

There are no conflicts to declare.

## Data Availability

No primary research results, software or code have been included and no new data were generated or analysed as part of this review.
